# Pediatric Extracorporeal Membrane Oxygenation (ECMO) Transport Safety—Regional and National Experiences and Literature Review

**DOI:** 10.3390/jcm15030925

**Published:** 2026-01-23

**Authors:** Jowita Rosada-Kurasińska, Bartłomiej Kociński, Anna Wiernik, Marcin Gładki, Mateusz Puślecki, Piotr Ładziński, Mark T. Ogino, Alicja Bartkowska-Śniatkowska

**Affiliations:** 1 Department of Paediatric Anaesthesiology and Intensive Therapy, Poznan University of Medical Sciences, 61-701 Poznań, Poland; 2 Department of Paediatric Cardiac Surgery, Poznan University of Medical Sciences, 61-701 Poznań, Poland; 3 Department of Medical Rescue, Poznan University of Medical Sciences, 61-701 Poznań, Poland; 4 Division of Neonatology, Children’s Hospital of Philadelphia, Philadelphia, PA 19104, USA

**Keywords:** extracorporeal membrane oxygenation, transport, pediatrics, acute respiratory failure

## Abstract

**Background/Objectives:** Venovenous extracorporeal membrane oxygenation (VV ECMO) supports reversible respiratory failure when mechanical ventilation fails. Technological advances and specialized teams now enable ECMO initiation at referring centers, even for high-risk transports. This study aimed to evaluate the safety of pediatric patients on ECMO support during medical transfer, based on a single-center experience and a systematic review of the literature. **Methods**: A retrospective analysis was conducted on all pediatric patients supported with ECMO transferred from regional hospitals to our university hospital (January 2023–September 2025), focusing on transport-related mortality and morbidity. We also performed a systematic review of original articles (2015–2025) using the PubMed, Embase, and Cochrane databases. **Results:** Fourteen critically ill children with a median age of 16 months (range: 2 months to 11 years) and acute respiratory failure were transferred to our hospital’s Intensive Therapy Unit. All transported patients in the local cohort were supported with VV ECMO. Transport distances ranged from 5 to 520 km (median: 151 km). No mortality or serious adverse events occurred during transfer. Two technical issues were noted. In the systematic review, 14 articles met the inclusion criteria, reporting a total of 900 transfers, mainly primary ECMO initiations (779–86.6%). The number of ground transports was 337, which accounted for 37.4%. Adverse events were reported in 252 out of 900, which was 28%. One death during transport was reported (mortality: 1‰). **Conclusions:** All transports were safely performed by our experienced multidisciplinary mobile ECMO team. Both our experience and literature review confirmed low mortality in pediatric ECMO transport, despite potential life-threatening adverse events.

## 1. Introduction

Extracorporeal membrane oxygenation (ECMO) techniques are successfully utilized in pediatric cardiac surgery units. Over recent years, there has been increasing interest in these methods among anesthesiologists working in pediatric intensive care units. Four ECMO support modes are commonly used and defined in the current nomenclature: venoarterial (VA) for cardiac failure, venovenoarterial (VVA) for cardiorespiratory failure, venovenous (VV) for respiratory failure, and venopulmonary (VP) for right ventricle/respiratory failure [1]. ECMO is not a treatment for the lungs but maintains life-sustaining support when lung function is so compromised that adequate arterial oxygenation and/or carbon dioxide elimination cannot be maintained. Importantly, ECMO should be considered in patients with diagnosed or suspected reversible pathologies for whom the risks of ECMO are outweighed by the risks of withholding such support. The decision to initiate ECMO should weigh the potential benefits against the risks and rely on multidisciplinary expertise [2].

Patients ideally should be transported to ECMO centers before they become unstable and impossible to transfer by conventional means. High-volume centers are associated with better survival outcomes. However, in many cases, the clinical course is unpredictable, and patients may deteriorate rapidly [3]. The first successful transport of a patient on extracorporeal membrane oxygenation (ECMO) was reported in 1986 by Cornish et al. [4]. Since then, numerous ECMO transports have been described, including those for neonates, pediatric patients [5,6], and adults [7]. Mobile ECMO for pediatric patients combines the challenges of hemodynamic management in low-weight patients with the technical complexities of transport.

The main objective of our study was to assess the safety of pediatric patients supported with VV ECMO who were transferred from regional hospitals to an ECMO center, based on our single-center experience and a systematic review of previously published clinical reports.

It should be emphasized that pediatric VV ECMO transport data are scarce, heterogeneous, and poorly standardized; moreover, most studies mix ECMO modes (e.g., VV and VA), which limits direct comparability to our pediatric VV-specific experiences, and this study aims to address this gap in the literature.

It is noteworthy that our team is currently the only one in Poland (38 million inhabitants) offering this highly specialized primary ECMO transport service for pediatric patients.

## 2. Materials and Methods

According to the rules of the Local Bioethical Committee of Poznan University of Medical Sciences, ethical approval is not required for retrospective studies based on documentation review and literature research; therefore, no formal ethical approval was necessary (decision number 418/25). All data processing was conducted in compliance with the EU General Data Protection Regulation (GDPR) and Polish national law. Pseudonymized datasets were used exclusively within the hospital as data controller, with no direct access granted to journals or external reviewers.

We analyzed the safety of pediatric patients supported with VV ECMO who were transferred from regional hospitals to our intensive therapy unit between January 2023 and September 2025. All consecutive pediatric patients transported on ECMO during the study period were included; no eligible cases were excluded. Missing data were rare, and analyses were performed using complete cases. The analysis included basic demographic variables, indications for ECMO support, vasopressor requirements, clinical diagnoses, clinical outcomes, ECMO types, transfer details, and complications. Retrospective data were collected from our Hospital Information System (HIS). Our primary focus was on transport safety, including mortality, morbidity, and other adverse events. Transport complications were classified according to the risk category system published by Fletcher-Sandersjöö et al. [8]. The ELSO guidelines were used to categorize ECMO transport as primary or secondary [1]. Primary transport refers to situations where the transport team performs cannulation for ECMO support at the referring hospital and then transfers the patient to the ECMO center. All patients were supported with VV ECMO using the percutaneous cannulation of the internal jugular vein with a single double-lumen cannula (Avalon Elite, Getinge AB, Gothenburg, Sweden). Two-dimensional ultrasonography-guided vascular puncture, guidewire insertion, and cannula positioning were performed. A mobile ECMO console certified for ground transport (Xenios console 1.0, Xenios AG, Heilbronn, Germany) equipped with diagonal magnetic pumps and polymethylpentene (PMP) NovaLung ECMO oxygenators (Xenios AG) was used in all cases.

### Statistical Analysis

Descriptive data for normally distributed continuous variables were presented as means  ±  standard deviations, and other distributions were presented as medians (interquartile ranges [IQRs]). Categorical variables were presented as percentages and numbers.

A systematic review of the Medline, Embase, and Cochrane databases from January 2015 to September 2025 was conducted to identify articles reporting the primary VV ECMO transportation of pediatric patients. Studies were included if they reported medical or technical complications during interhospital transfers of pediatric patients receiving extracorporeal support. The review included original papers published before the end of September 2025. The search strategy employed the following MeSH (Medical Subject Heading) terms and Boolean operators: (“Extracorporeal Membrane Oxygenation”[MeSH] OR “ECMO”[MeSH]) AND (“Transportation”[MeSH] OR “pediatric patient transfer”[MeSH]) AND “child”[MeSH Terms].

Additional inclusion criteria were articles published in English after 2015, in the form of randomized controlled trials (RCTs), meta-analyses, practice guidelines, or reviews. Studies focusing on adult patient transfers, transports solely for the need of ECMO support, and single-case reports were excluded. Remaining articles were independently assessed for eligibility by three investigators, and only manuscripts approved by at least two investigators were included. The review process followed the PRISMA (Preferred Reporting Items for Systematic Reviews and Meta-Analyses) guidelines [9]. When extracting data from eligible studies, we attempted to obtain pediatric-only outcomes wherever possible; however, many reports provided only aggregated results across mixed-age cohorts.

From the selected articles, the following data were extracted: the country where the study was performed, study start and completion years, reported study duration (in months), number of transports, cannulation type (VA or VV ECMO), mode of transport, transport classification (primary or secondary), occurrence of deaths or adverse events, minimal and maximal transfer distances, and number of personnel on the mobile ECMO team. The ECMO mode (VV vs. VA) was recorded for each study. Where sufficient data were available, we performed subgroup analyses restricted to VV cohorts; otherwise, studies with mixed modes were pooled, and mode-related differences were considered qualitatively when interpreting results.

Although no universally accepted scoring system exists for the evaluation of case series involving patients on ECMO support, we established a predefined assessment method for all included studies. This approach was based on the methodology of previously published reports [10]. Each study was systematically rated, and an aggregate score was calculated to estimate the overall data quality (Table S1).

The rate of survival with the standard error and 95% confidence interval (CI) were derived for each trial. The Cochrane Q-statistic test was performed to assess the statistical heterogeneity, and the I2 statistic was used to estimate the extent of variability attributable to statistical heterogeneity in the results between the included studies. Assessment of asymmetry (presence of publication bias) was performed using a funnel plot and Egger’s test. The pooled-effect sizes were calculated using a random-effects model based on the heterogeneity across studies. We performed a random-effects meta-analysis, as substantial clinical and methodological heterogeneity was expected across studies with respect to patient age, ECMO mode (VV vs. VA), and transport practices.

All the statistical analyses were carried out using PQStat 1.8.6 (PQStat Software, Poznan, Poland) or Statistica 14 (TIBCO Software Inc., Palo Alto, CA, USA).

## 3. Results

Fourteen critically ill children supported with VV ECMO (3 girls and 11 boys), aged 2 months to 11 years (median age: 16 months; IQR: 13–36 months), were transported from regional hospitals to our ECMO center. Characteristics of the patients, including those with hospital mortality, are presented in Table 1.

The primary indication for cannulation was pediatric acute respiratory distress syndrome (PARDS) with failure of conventional therapy. The mean ICU stay before the ECMO decision was 3.9 days. Infectious pathogens identified included respiratory syncytial virus (RSV) in four patients, Influenza A in one, COVID-19 in one, and Streptococcus pneumoniae in three; in five cases, the pathogen remained unidentified. Eleven patients required vasopressors before cannulation. At transport initiation, all patients experienced an increase in arterial blood pressure, necessitating vasopressor dose reductions; vasopressors were discontinued in six patients and started in one, and vasodilator therapy was required in one case.

Fluid therapy, including additional boluses for relative hypovolemia, was administered to all patients. Three required tracheobronchial drainage. The mean time from receiving the referral hospital report to departure was 4.38 h. The median transport distance was 151 km (range: 5 to 520 km). The mean ECMO support duration was 10.21 days (range: 4–18 days). There was 100% survival during transport, and 11 patients (78.6%) survived to hospital discharge. In one of the patients, already at our ECMO center, VV ECMO was converted to V+V ECMO (two serial membrane lungs in VV ECMO configuration). According to the ELSO nomenclature, V+V ECMO (two serial membrane lungs) refers to a VV ECMO setup in which blood from the patient passes through two membrane oxygenators arranged in series to provide enhanced respiratory support [1]. Two technical problems occurred during transport, classified as category III risk—no immediate risk of morbidity or mortality but requiring attention. In one case, the oxygen supply approached critical levels; coordination with Poznan Emergency Medical Services and the Emergency Communication Centre enabled replenishment at a nearby hospital before transport continued. The patient arrived stable for further care at the home facility. In another case, there was a problem with the heater–cooler, and the water supply was depleted. The issues were solved as they arose. Both events were promptly recognized through continuous monitoring of gas supply pressures and circuit temperature alarms and were managed by switching to a backup oxygen source and adjusting the heater settings without clinical deterioration of the patients. These incidents led us to reinforce pre-departure checks of gas reserves and to introduce explicit temperature trend verification onto our transport checklist. Details are provided in Table 2.

All transports were conducted only by ground with a dedicated mobile ECMO team. Therefore, differences in transport distances and modality means in comparisons with studies involving air transport, should be interpreted with caution.

## 4. Literature Review

**Figure 1:**
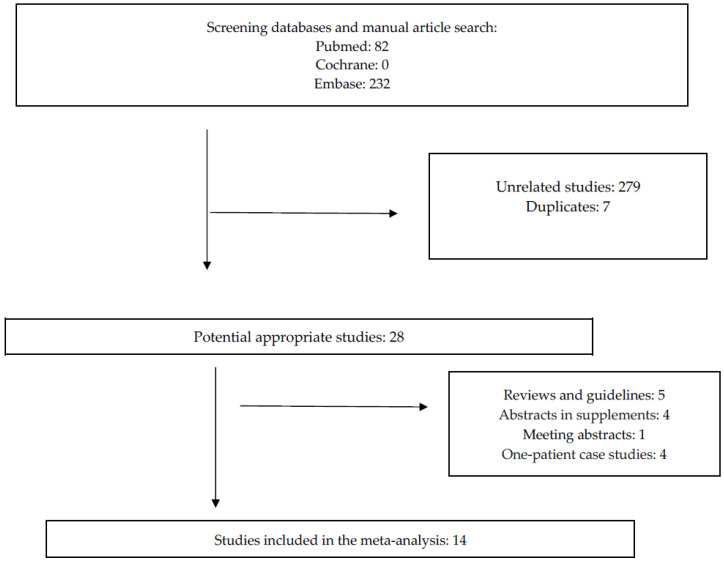
Research flowchart.

A total of 314 publications were initially identified, but 279 were excluded at the preliminary screening because they did not meet the inclusion criteria. These excluded studies either involved only adult patients, described transfers of critically ill children without ECMO support, reported solely the need for ECMO, were case studies as clearly indicated in the title, or were not published in English. Duplicate manuscripts were also excluded. Twenty-eight full-text articles met the inclusion criteria and underwent review. Following detailed analysis, an additional five papers were excluded due to their report types—they were reviews or guidelines without ECMO transport data such as numbers of transfers or distances. Four more publications accepted as abstract supplements and one meeting abstract were excluded, as were four single-patient case reports. Ultimately, 14 articles were included for analysis (Figure 1), illustrating the scarcity of data on pediatric ECMO transport.

The included studies reported a total number of 900 transports. Most were single-center experiences; only one publication described over 100 transports [8], and four reported over 50 [11,12,13,14]. Transfers were predominantly primary (779 (86.6%)), and a total of (337 (37.4%)) ground-based transfers were performed. Various transport modes were used, including ambulance, helicopter, airplane, and hybrid transfers. Both VV and VA ECMO patients were included in nearly all studies. Four reports focused exclusively on VA ECMO [5,15,16,17]; none reported only VV ECMO transports. The median monthly transport volume was 0.31. The median minimum transfer distance was 70 km, and the median maximum transfer distance was 447 km. Team sizes ranged from two to seven members (median: four). Only one death during transport was documented in the included studies (mortality: 1‰) [8].

The main indications for ECMO support were pediatric ARDS and cardiac failure. The published literature does not generally distinguish indications separately for VA versus VV ECMO support. Percutaneous cannulation was the predominant technique for both modes; one study reported exclusively surgical cannulation [14]. Reported adverse events during transport included transient hemodynamic instability, technical issues such as circuit alarms or flow reductions, and hypotension. A list of the included studies is presented in Table 3.

In our analysis, only two deaths out of 900 pediatric transfers were reported from 2015 to 2025 [5,8]. Among these, one death was associated with cannulation but not directly related to transport, and one was directly linked to the medical transfer to the referral ECMO center.

It is also possible that transport programs with less favorable or more complication-prone experiences are underrepresented in the published literature, leading to a potential bias toward reporting more successful ECMO transports.

**Figure 2:**
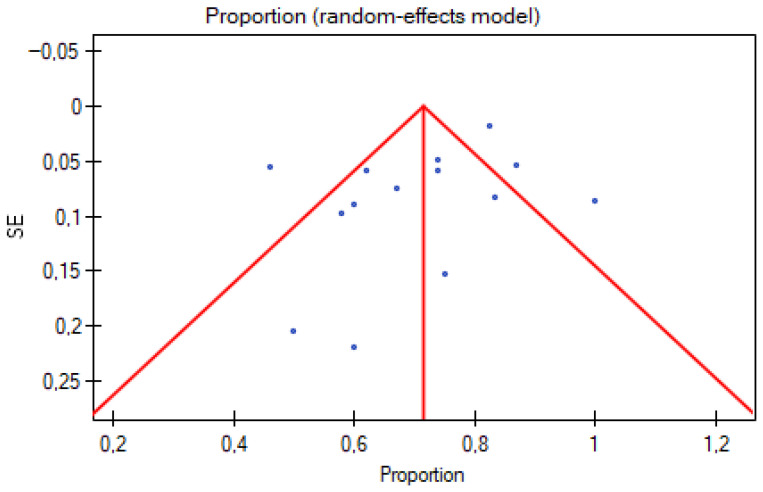
Funnel plot of publication bias.

No publication bias was detected (Egger’s test: b = −1.690, *p* = 0.102; funnel plot asymmetry not evident) (Figure 2), but significant heterogeneity was present (Q = 70.10, *p* < 0.001, I^2^ = 81.5%) (Table 4).

The high I^2^ value indicated substantial heterogeneity across studies, and this was likewise supported by the Q statistic.

The pooled survival rate for all fourteen studies was 72% (95%CI: 64–79%).

**Figure 3:**
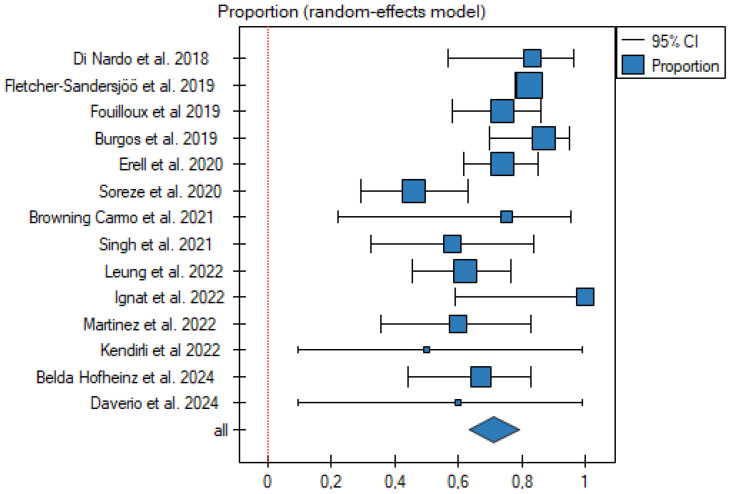
Meta-analysis summary. Pooled proportion of survivors in published pediatric ECMO transport studies. Each square represents the estimated proportion of survivors in an individual study, with the size of the square being proportional to the study weight in the random-effects model. Horizontal lines indicate 95% confidence intervals (CIs). The diamond at the bottom represents the pooled proportion with its 95%CI. The vertical dashed line indicates the overall pooled estimate [5,6,8,11,12,13,14,15,16,17,18,19,20,21].

A meta-analysis of the number of patients who ultimately survived thanks to ECMO support is presented in Figure 3 and Table 5.

**Table 5:** Meta-analysis summary.

Publication	n	Proportion	SE	−95%CI	+95%CI
Di Nardo et al., 2018 [18]	17	0.83	0.08	0.57	0.96
Fletcher-Sandersjöö et al., 2019 [8]	358	0.83	0.02	0.78	0.86
Fouilloux et al. 2019 [11]	42	0.74	0.06	0.58	0.86
Burgos et al., 2019 [19]	35	0.87	0.05	0.70	0.95
Erell et al., 2020 [12]	59	0.74	0.05	0.62	0.85
Soreze et al., 2020 [13]	37	0.46	0.06	0.29	0.63
Browning Carmo et al., 2021 [15]	6	0.75	0.15	0.22	0.96
Singh et al., 2021 [16]	15	0.58	0.10	0.32	0.84
Leung et al., 2022 [14]	42	0.62	0.06	0.46	0.76
Ignat et al., 2022 [20]	7	1.00	0.09	0.59	1.00
Martinez et al., 2022 [5]	18	0.60	0.09	0.36	0.83
Kendirli et al. 2022 [17]	3	0.50	0.20	0.09	0.99
Belda Hofheinz et al., 2024 [6]	26	0.67	0.08	0.44	0.83
Daverio et al., 2024 [21]	3	0.60	0.22	0.09	0.99
**All**	**668**	**0.72**	**0.04**	**0.64**	**0.79**

## 5. Discussion

The most recent literature review on mobile ECMO in children was conducted by Mendes et al. [10]. They reported single-center data on complications and mortality associated with transportation of patients on ECMO support. Additionally, they searched multiple databases for articles on mortality in patients transferred on ECMO support up to November 2012. Their results were analyzed separately for patients under and over 12 years old. They concluded that patient transfer to a referral institution while on ECMO support appears to be safe and does not significantly increase the mortality risk for ECMO patients [10]. Erell et al. [12] also conducted a small literature review on ECMO-supported transport, including publications from 1991 to 2019. In recent years, no meta-analyses focused exclusively on pediatric ECMO patients have been published.

In this study, the interhospital ground transport of pediatric patients on VV ECMO proved feasible and was associated with no transport-related mortality and only infrequent serious complications, whereas the systematic review of 900 transports revealed a low yet notable incidence of adverse events. Together, these findings indicate that pediatric ECMO transport could be conducted safely by experienced multidisciplinary teams, but important clinical risks persist and must be carefully anticipated.

### 5.1. Hub and Spoke

To improve outcomes and optimize healthcare resources, consolidation of ECMO support in high-volume centers has been proposed [22]. The “Hub and Spoke” ECMO model is a regional (though it can be national or international) healthcare network designed to provide emergent extracorporeal membrane oxygenation (ECMO) support to patients with severe, life-threatening cardiorespiratory conditions at peripheral (spoke) hospitals, followed by safe transport to a high-volume ECMO center (hub) for definitive care. Such systems require efficient coordination of ECMO procedures. The concept of “Hub and Spoke” was introduced by Combes et al. [22], providing practical guidance on patient selection, initiation, management, cannulation, and weaning from VV ECMO for adults with respiratory failure.

The “Hub and Spoke” ECMO model for neonates with life-threatening respiratory conditions was described by Padalino et al. [23]. They concluded that the “Hub and Spoke” ECMO model for neonates is effective and successful. It is clear that accurate patient selection, an experienced ECMO transport team, specialized protocols and checklists, and close cooperation between hub-and-spoke centers are essential for success [23]. Additionally, Fichera et al. [24] evaluated the clinical outcomes of emergent ECMO implantation in newborns with life-threatening meconium aspiration syndrome (MAS) using a hub-and-spoke model in peripheral hospitals. Our regional ECMO program functions as a classic hub-and-spoke network, with our center serving as the hub receiving referrals and providing mobile ECMO support to surrounding spoke hospitals, like models described in previous reports.

### 5.2. Center Experience, Patient Volumes, and Results

The use of extracorporeal membrane oxygenation for severe acute respiratory failure in adults is increasing due to recent technological advances. While ECMO for respiratory failure in pediatrics and neonates has shown overall growth in specialized centers, pediatric ECMO volumes have remained stable, and neonatal cases have declined in the past several years. ECMO is a complex, high-risk, and costly modality, influenced both by the patient’s clinical severity and the numerous devices that must be mobilized alongside the patient. Currently, ECMO should be performed in centers with sufficient experience, patient volumes, and expertise to ensure safe application [23]. There are no published data defining the minimum number of transports that a mobile ECMO team should perform annually. Regarding adults, a consensus statement in the ELSO guidelines recommends a minimum of 20 cases per year [1]. The ELSO has also defined clinical criteria for patient selection for ECMO support, although robust data on the indications, timing, and mode of ECMO in children remain insufficient [1]. For neonatal and pediatric patients, definitive data are rare; however, a similar volume threshold has been recommended to improve survival rates compared with low-volume intensive care units [25]. Gonzalez et al.’s large analysis of 4546 ECMO-supported patients identified a threshold of 30 annual cases wherein higher volumes significantly improved mortality outcomes [26]. Below this cutoff, pediatric ECMO survival rates showed no difference between low-volume centers or between pediatric versus non-pediatric facilities [26]. Notably, the study did not address ECMO transport volumes. In summary, the volume–outcome relationships referenced in this discussion are derived predominantly from adult ECMO cohorts and may not be directly generalizable to pediatric transport; pediatric-specific data remain limited and are supported mainly by a few observational studies [25,26,27].

In our study, only 14 patients were transported between hospitals over the past three years. However, our expertise has been developed within the cardiac surgery department, focusing on comprehensive extracorporeal circulation management, including monitoring, cannulation of peripheral veins and arteries, teamwork, and communication during transport. We have established safety measures and protocols for intrahospital transport and have achieved favorable outcomes despite the relatively low number of transports.

In our analysis, the median number of transfers per month was 0.41. Our results are comparable to those reported in the reviewed literature. Data concerning adults, however, differ significantly. Puślecki et al. [7], in their meta-analyses, describe a much larger number of transports and greater experience with adult patients, even suggesting that ECMO transport in pediatric patients is relatively infrequent. This assertion is not supported by our experience or by the literature reviewed [7].

It should be emphasized that our cohort consisted exclusively of pediatric patients supported with VV ECMO for respiratory failure, whereas many published transport series include a substantial proportion of VA ECMO cases; therefore, our findings should be interpreted as most applicable to VV ECMO transports in children.

### 5.3. Transportation Definition, ELSO Guidelines

Transporting a patient on ECMO support combines the challenges of the traditional transport of critically ill, mechanically ventilated patients receiving drug infusions with the additional complexity of managing ECMO. Transporting patients on ECMO is highly demanding due to this complexity. The concept of cannulating a patient at the referring hospital and transporting them on ECMO was first described by Bartlett et al. [28]. Later, Cornish developed the practice further by introducing transport with a mobile ECMO system, performing cannulation at the referring hospital, and then transporting the patient on ECMO support to the ECMO center [4].

According to ELSO definitions, patients cannulated at a referring hospital by the ECMO transport team and then subsequently returned to the ECMO center are classified as primary transports [1]. Patients already placed on ECLS at an outside hospital and subsequently transferred by a transport team are classified as secondary transports. It is also important to note that patients on ECLS may be transported interhospital—for transfer between hospitals—or intrahospital—for therapeutic or diagnostic procedures within the same center [1].

We have three transport options available for ECMO support: ground ambulance, helicopter, and fixed-wing aircraft. According to ELSO guidelines, ground transport is recommended for distances up to 400 km (250–300 miles), helicopter transport is suitable for distances up to 650 km (300–400 miles), and fixed-wing aircraft can be used for any distance [1]. Transport vehicles must have an adequate electrical supply to power the ECMO pump, heater, and all other electrical equipment used during transport. Additionally, the oxygen supply must be sufficient for the entire duration of the transport.

Our chosen mode of transport was ground transport using a container ambulance registered as a truck due to its weight. This choice is dictated by the patient’s condition and the requirement for intensive care during the critical hours of VV ECMO system operation. The device we use (Xenios 1.0) comes with recommended pediatric sets but is not certified for air transport. Conversely, the Cardiohelp (Getinge) System is certified for air transport but does not have pediatric sets available [29].

Our ECMO team does not maintain regular ECMO shifts. Upon request by the ECMO transport coordinator, a team of trained specialists is voluntarily assembled, including one–two pediatric anesthesiologists, one–two perfusionists, a cardiac surgeon (if complications during cannulation are anticipated), and an ambulance driver. Each team member has a designated role in ECMO management, as described by Padalino et al. [23]. Although our ECMO team operates on a voluntary basis, the mean response time from the call to departure was 4.38 h (range: 1.5–9 h), comparable to other published data, such as those by Belda Hofheinz et al. and Daverio et al., who reported response times ranging from 2 to 8 h [6,21]. Our team is a highly skilled and specialized pediatric critical care transport team, organized and coordinated by the pediatric intensive care and cardiac intensive care units at Karol Jonscher University Hospital.

The decision to initiate VV ECMO was made by the intensive care physician at the referring center after confirming inclusion criteria based on the rapid deterioration of the patient’s condition. Necessary conditions included prior use of all available intensive treatments for acute respiratory failure, lack of improvement in gas exchange with worsening trends, a deteriorating overall prognosis, and exhaustion of conventional treatment options.

Published data indicated that ECMO transport teams mainly consisted of pediatric intensivists, pediatric surgeons, perfusionists, and nurses. Burgos et al. described the individual managing the ECMO machine not solely as a perfusionist but also as an ECMO specialist nurse [19]. This observation aligns with our team structure in Poland, where anesthesiology training includes intensive care specialization, allowing anesthesiologists to serve as intensivists.

### 5.4. Classification of Complication

Barrigoto et al. state that when ECMO-supported transport is performed by an experienced team, severe complications are not associated with increased morbidity and mortality. Their data, however, concern adult patients [30]. This does not exclude the possibility that high-risk and life-threatening events may still occur. Review articles mostly reported transient hemodynamic instability, technical issues such as circuit alarms or flow reductions, and hypotension as complications during transport. There was considerable variability in how complications during patient transport were defined across studies, complicating an accurate assessment of the problem’s true extent. In four previously published reports, authors Belda Hofheinz, Singh, Leung, and Fletcher-Sandersjöö used the following categorization for the risk stratification of complications [6,8,14,16]:

- Risk category I events: high risk for morbidity and mortality without response within seconds.
- Risk category II events: high risk for morbidity and mortality with no response within minutes.
- Risk category III events: in need of attention, with no risk to morbidity or mortality.
- Risk category IV events: low risk needed to be noted.

Categories I and II were observed in 11.5% of patients in Singh’s study and in 20% of events reported by Fletcher-Sandersjöö; however, these complications had no impact on mortality [8,16]. The only death reported by Fletcher-Sandersjöö during transport occurred in a patient on VV ECMO due to circulatory failure and cardiac arrest [8]. This death might have been prevented if the circulatory collapse had occurred in the ICU, where conversion to VA ECMO could have been performed [8]. Several pediatric and mixed-age transport series reported clinically significant complications, including category I-II events in 11.5–20% of transports and at least one transport-associated death in a VV ECMO patient. These data illustrate why ECMO transport has been considered operationally challenging and potentially high-risk. In Leung’s study, complications were quite common, occurring in 65.95% of transports [14]. Nevertheless, these issues were managed promptly and did not result in adverse outcomes. Specifically, 22 complications were classified as category I, 94 as category II, 64 as category III, and 9 as category IV [14]. Burgos et al. described complications in 40% of transports, with the most frequent being loss of tidal volumes (35%), equipment failure (20%), and climate or transport vehicle problems (15%) [19]. Belda Hofheinz et al. reported a 56.4% complication rate, mostly related to the means of transport [6]. Daverio also mentioned minor complications, such as the occurrence of differential hypoxemia and cannula displacement [21]. Fouilloux, Ignat, and Kendirli reported no transport-related complications [11,17,20]. Similarly, Erell et al. found no technical complications, major equipment malfunctions, or patient deaths during transport [12]. Martinez also reported no complications but did note one death related to cannulation [5]. Our study reflected the same trend, with only two technical problems occurring during transportation of patients with ECMO support to our center. According to the Fletcher-Sandersjöö classification, these complications were categorized as risk level III—no immediate risk of morbidity or mortality but requiring resolution.

Hemodynamic changes were also observed in our patients, necessitating the initiation or discontinuation of catecholamine therapy. These physiologic fluctuations during transport may reflect a combined effect of sedation, stress response, and patient positioning; however, the exact mechanism remains uncertain and warrants further investigation.

Burrell et al. reported that adult patients on VA ECMO required more transport interventions compared with those on VV ECMO [31]. However, Fletcher-Sandersjöö et al., based on their own data, found no evidence that VA ECMO itself increases the risk of complications [8]. Our analyzed studies also do not support this association. All our patients were managed using venovenous ECMO via percutaneous cannulation of the internal jugular vein with a single dual-lumen cannula. Proper positioning of the double-lumen cannula is challenging and requires significant expertise and technical skill. In the reviewed publications, most cannulations were for VA ECMO (673 (75%)). This predominance results not only from clinical indications—VV ECMO being implemented for respiratory failure without hemodynamic compromise, and peripheral VA ECMO for patients with hemodynamic compromise—but also from technical difficulties encountered in younger patients, as noted by Martinez et al. and Soreze et al. [5,13]. Technical demands contribute to the operational difficulty of pediatric ECMO transport. In Leung’s pediatric cohort, 41.2% of children on VV ECMO required conversion to VA, with nearly one quarter of conversions driven by cannulation-related technical challenges [14].

Part of the variability in the reported adverse event rates is probably attributable to differences in how complications were defined and documented across studies, such as what was categorized as a circuit-related versus hemodynamic event. This indicates that much of the observed heterogeneity arises from inconsistencies in the underlying literature rather than from the analytic approach used in this review. In many of the included studies, outcomes were reported for mixed neonate–pediatric cohorts without separate data for neonates versus older children; thus, some of the pooled estimates inevitably combine these age groups and limit age-specific interpretation.

### 5.5. Study Limitations

The available evidence in this systematic review/meta-analysis remains limited by several important factors. Firstly, there is limited data on the transport of pediatric patients with ECMO support. Accurate estimation of the number of ECMO transfers, deaths, or other incidents based on published reports was challenging because conventional transports were often included in the counts. Many studies included both adults and children, and some were meta-analyses without clear subgroup distinctions. Most studies lacked a scoring system to correlate expected mortality with actual outcomes. Large multicenter prospective studies were absent, and our study is also retrospective in design. Moreover, the included cohorts differed in patient ages (from neonates to adolescents), ECMO modes (mixed VV and VA populations), and transport modalities (ground, rotary-wing, and fixed-wing). This variability limits the strength and generalizability of the combined estimates and suggests that our conclusions are most appropriately applied to settings with similar case mixes, ECMO configurations, and transport practices. Additionally, heterogeneity in the definitions of adverse events complicates direct comparisons between studies. Data specifically focused on VV ECMO are scarce, as most studies report on mixed patient populations. Furthermore, the existing research primarily focuses on short-term survival, providing limited information on long-term outcomes. These limitations should be considered when interpreting our findings.

### 5.6. ECMO for Greater Poland

Promoting ECMO for severe respiratory/circulatory failure is clinically crucial in Poland. Effective resource utilization, following international standards, remains challenging due to the lack of systemic access and clear VV/VA ECMO epidemiological data [32].

Implementing ECMO in Poland faces challenges, particularly in pediatrics, due to absent systemic protocols, training, staffing, and ICU capacity. VV ECMO for respiratory failure develops in select centers, while VA ECMO relies on cardiac surgery departments. Moreover, land and air transport solutions to specialized centers remain lacking.

The pediatric mobile ECMO program is part of a larger initiative, “ECMO for Greater Poland,” established in 2016 to serve a population of 3.5 million inhabitants. In 2016 in Wielkopolska, thanks to the employees and departments of Poznan University of Medical Sciences, the “ECMO for Greater Poland” program was created [32]. The overarching goal of the program was to popularize extracorporeal techniques for the critically ill of the 3.5 million population of the Greater Poland Region. The aim of the “ECMO for Greater Poland” program was to create system-wide procedures for the identification of potential candidates for the use of extracorporeal perfusion support and transport to specialized medical centers to implement support and conduct it at the highest level.

The number of centers providing transport with mobile ECMO teams has increased, and such transports are now widely performed across Europe [13]. In Poland, we are still the only center conducting transports of pediatric patients on ECMO support. Over the past two years, we have treated 15 pediatric patients with VV ECMO support in our Pediatric Intensive Care Unit. In the pediatric cardiac surgery unit, an average of 12 VA ECMO procedures is performed annually. Currently, there is no systemic framework or national guidelines in Poland specifically addressing pediatric ECMO. Consequently, the next phase of our work will focus on developing comprehensive national guidelines for the application of VV ECMO in pediatric patients. It is also worth noting that, in recent years, there have been no publications describing pediatric ECMO transports either from Poland or from other former Eastern European countries. Prospective multicenter studies using harmonized reporting standards are needed to better quantify transport-related risks and identify modifiable factors that can further improve the safety of pediatric ECMO transport.

## 6. Conclusions

In our experience and based on the reviewed literature, transporting children on ECMO support was safe when both the institutional ECMO program and conventional transport centers had substantial experience. Such transport can be conducted safely by a well-trained, dedicated ECMO team working closely together. ECMO transport carries inherent risks related to the transport process itself, as well as complications associated with ECMO support and patients’ underlying medical conditions. These insights inform practice for pediatric VV ECMO transport teams by emphasizing the value of specialized training and institutional expertise.

## Figures and Tables

**Table 1:** Demographic and clinical characteristics.

Characteristics	Overall (n = 14)
Age	2 months–11 years (median: 16 months)
Weight (kg)	4.3–20 kg (mean: 10.9 kg ± 4.2)
Sex	
Male (n (%))	11 (78.6%)
Female (n (%))	3 (21.4%)
Need for vasopressors before cannulation (n (%))	11 (78.6%)
Time in ICU before cannulation (days) (mean)	3.86 ± 2.63
Primary diagnosis	
Respiratory (n (%))	14 (100%)
Pathogens responsible for infection	
RSV (n (%))	4 (28.6%)
Influenza A (n (%))	1 (7%)
COVID-19 (n (%))	1 (7%)
Streptococus pneumoniae (n (%))	3 (21.4)
Not identified (n (%))	5 (36%)
Distance from reporting center (km)	5–520 km (median: 151 km)
Departure time from moment of receiving report (h)	1.5–9 h (mean: 4.38 h ± 2.02)
Transport type	
Primary (n (%))	14 (100%)
Transport mode	
Ground (n (%))	14 (100%)
Time on ECMO support (days)	4–18 (mean: 10.21 ± 3.7)
Survivors (discharged home) (n (%))	11 (78.6%)
Technical problems (n (%)) Risk category 3 events	2 (14.3%)

ICU: intensive care unit, RSV: respiratory syncytial virus, COVID-19: coronavirus disease 2019; ECMO: extracorporeal membrane oxygenation.

**Table 2:** Summary of all patients transferred with VV ECMO support.

No	Sex ^1^	Age ^2^	Body Mass (kg)	Departure Time from Moment of Receiving Report (h)	ECMO Transportation Distance (km)	Indication	Vasopressors Before Cannulation	Vasopressors During Transport	Deaths or Incidents
1	M	14 m	7.5	5	205	PARDS	yes	yes	none
2	F	11 y	20	4	135	PARDS	yes	yes	none
3	M	14 m	10	6	151	PARDS	no	no	none
4	M	18 m	9.1	5	151	PARDS	no	no	none
5	M	4 y	15	1.5	520	PARDS	yes	yes	problem with oxy supply
6	M	4 y	13	3	5	PARDS	yes	no	none
7	F	21 m	9	3	151	PARDS	yes	no	none
8	M	11 m	6.7	7	135	PARDS	yes	no	none
9	M	2 m	4.3	9	143	PARDS	no	yes	none
10	F	13 m	9	3	151	PARDS	yes	no	none
11	M	11 m	8	3.2	151	PARDS	yes	no	none
12	M	14 m	11	6	151	PARDS	yes	yes	none
13	M	24 m	15	1.5	5	PARDS	yes	yes	none
14	M	3 y	15	4	151	PARDS	yes	no	ECMO heater failure

^1^ M—male, F—female; ^2^ m—months, y—years; ECMO: extracorporeal membrane oxygenation; PARDS: pediatric acute respiratory distress syndrome; oxy: oxygen.

**Table 3:** List of included studies.

Publication	Center/Country	Reporting Period	No of Transfers	ECMO Team	Patient Age Median/Range	Cannulation Type	VV	Ground (%)	Air (%)	Adverse Events	Deaths	Transport Type Primary (%)	Distance (km) (Min)	Distance (km) (Max)
Di Nardo et al., 2018 [18]	Italy	2013–2018	20	7	2.1–55.7 days	VA/VV	6	70	30	25%		100	n/a	200–400
Fletcher-Sandersjöö et al., 2019 [8]	Sweden	1996–2017	434	2	n/a ^1^	VA/VV	163	30	70	30%	1	89	217–284	640–764
Fouilloux et al., 2019 [11]	France	2006–2016	57	4	2.89 months	VA/VV	2	38.6	8.7	None		100	1	350
Burgos et al., 2019 [19]	Sweden	1997–2007, 2008–2018	40	3	1 day	VA/VV	2	20	72.5	35%		97.5	428	1381
Erell et al., 2020 [12]	Israel	2003–2018	80	2	2 days	VA/VV	5	98.75		None		100	60	126
Soreze et al., 2020 [13]	France	2014–2019	80	3	n/a ^2^	VA/VV	23	70	30	39%		100	49	329
Browning Carmo et al., 2021 [15]	Australia	2015–2021	8	5	15 months	VA	0	25	75	None		100	26	2000
Singh et al., 2021 [16]	USA	1998–2017	26	3–4	15.5 days	VA	0	20	80	11.5%		61.5	72	447
Leung et al., 2022 [14]	Canada	2004–2018	68	4–6	20.4 months	VA/VV	17	1.5	98.5	65.95		15	298	1068
Ignat et al., 2022 [20]	UK	2018–2020	7	n/a	10 years	VA/VV	2	n/a	n/a	None		100	n/a	n/a
Martinez et al., 2022 [5]	Argentina	2011–2020	30	3–4	24 months	VA	0	56	44	None	1 during cannulation	100	n/a	1708
Kendirli et al., 2022 [17]	Turkey	2016–2020	6	4	112 months	VA	0	0	100	None		50	407	955
Belda Hofheinz et al., 2024 [6]	Spain	2018–2023	39	4–6	1.24 months	VA/VV	6	n/a	n/a	56.4%		100	70	393.25
Daverio et al., 2024 [21]	Italy/Spain	2020–2021	5	6–7	42–144 months	VA/VV	1	80	20	40%		100	8	390

^1^ The data presented include only the counts of pediatric patients (n = 150) and neonates (n = 284). ^2^ The data presented include only mean (days) 834 ± 1429. n/a not available.

**Table 4:** Heterogeneity test.

Heterogeneity Test	
Q statistic	70.097774
Degrees of freedom	13
*p*-value	<0.000001

## Data Availability

The datasets used and/or analyzed during the current study are available from the corresponding author upon reasonable request.

## Supplementary Materials

The following supporting information can be downloaded at https://www.mdpi.com/article/10.3390/jcm15030925/s1, Table S1: Quality evaluation of the manuscripts.

## Abbreviations

The following abbreviations are used in this manuscript:

GDPR	General Data Protection Regulation
ECMO	Extracorporeal membrane oxygenation
ELSO	Extracorporeal Life Support Organization
VV	Venovenous
VA	Venoarterial
VVA	Venovenoarterial
VP	Venopulmonary
V+V ECMO	Two serial membrane lungs
HIS	Hospital information system
MeSH	Medical subject heading
RCTs	Randomized controlled trials
PRISMA	Preferred Reporting Items for Systematic Reviews and Meta-Analyses
PMP	Polymethylpentene
ICU	Intensive care unit
PARDS	Pediatric acute respiratory distress syndrome
RSV	Respiratory syncytial virus
COVID-19	Coronavirus disease 2019
MAS	Meconium aspiration syndrome
ECM	Extracorporeal circulation
ECLS	Extracorporeal life support
