# Supplementary material for: Pediatric Extracorporeal Membrane Oxygenation (ECMO) Transport Safety—Regional and National Experiences and Literature Review
Source: J Clin Med. 2026 Jan 23;15(3):925. doi: 10.3390/jcm15030925 (PMC12897960; doi:10.3390/jcm15030925)
Supplement: Supplementary file 1 [file jcm-15-00925-s001.zip › jcm-4055179-supplementary.pdf]

Table S1: Quality evaluation of the manuscripts

Author	Year of publication	Clear aim	Multicenter	Clearly respiratory support	Etiology	Age	Pediatrics / adults clear division	Severity score	Gender	Weight or BMI	Transportation method	Mission time	Mission distance	Pre-ECMO MV time	Pre-ECMO pH	Pre-ECMO P/F ratio	Pre-ECMO $PaCO_2$	Configuration	Time on ECMO support	ICU-LOS	Hospital-LOS	Clear endpoint of survival	Consecutive cases	Intercurrences description	Final punctuation
Di Nardo et al. 2018	2018	1	0	1	1	1	1	0	1	1	1	1	1	1	1	1	1	1	0	0	0	1	1	1	7,8
Fletcher-Sandersjöö et al. 2019	2019	1	0	0	0	1	1	0	0	0	1	1	1	0	0	0	0	1	0	0	0	1	0	0	3,04
Fouilloux et al 2019	2019	1	0	0	0	1	1	0	1	1	1	1	1	0	0	0	0	1	0	0	0	1	1	1	5,65
Burgos et al. 2019	2019	1	0	0	0	1	1	0	1	1	1	1	1	0	0	0	0	1	1	0	1	1	1	1	6,52
Erell et al. 2020	2020	1	0	1	1	1	1	1	0	0	1	1	1	0	0	1	0	1	1	0	0	1	0	1	5,65
Soreze et al. 2020	2009	1	0	1	1	1	1	1	1	1	1	1	1	1	1	1	1	1	1	0	0	1	1	1	8,69
Browning Carmo et al. 2021	2021	1	0	0	0	1	1	0	0	1	1	1	1	0	0	0	0	1	0	0	0	1	1	0	4,34
Singh et al. 2021	2021	1	0	0	0	1	1	0	1	1	1	1	1	0	0	0	0	1	0	0	0	1	1	0	4,78
Leung et al. 2022	2022	1	0	0	0	1	1	0	1	1	1	1	1	1	1	1	1	1	0	1	0	1	1	1	7,39
Ignat et al. 2022	2022	1	0	1	1	1	1	0	1	1	1	0	0	0	0	0	0	1	1	0	0	1	1	1	5,65
Martinez et al. 2022	2022	1	0	1	1	1	1	1	1	1	1	1	1	1	1	1	1	1	1	0	0	1	1	1	8,69
Kendirli et al 2022	2022	1	0	0	0	1	1	0	1	1	1	1	1	0	0	0	0	0	1	0	0	1	1	1	5,21
Belda Hofheinz et al. 2024	2024	1	0	0	1	1	1	1	1	1	1	0	1	1	1	1	1	1	1	1	0	1	1	1	8,26
Daverio et al. 2024	2024	1	0	1	1	1	1	1	1	1	1	1	1	1	0	1	0	1	1	0	0	1	1	1	7,82

The number one indicates the presence of the characteristic analyzed.
Final punctuation is the sum of the points (0 or 1) divided by 23 (total of characteristics analyzed) times 10.
ECMO denotes Extracorporeal membrane Oxygenation; BMI denotes body mass index; LOS denotes Length-of-stay; and ICU denotes Intensive Care Unit.
